# Factors influencing dietary patterns among the youth from higher educational institutions in India

**DOI:** 10.3389/fnut.2023.1134455

**Published:** 2023-03-28

**Authors:** Sudhir K. Soam, B. S. Yashavanth, Thammi Raju Dhumantarao, Balakrishnan Maruthamuthu, Raghupathi Balasani, S. Rakesh

**Affiliations:** ICAR-National Academy of Agricultural Research Management (NAARM), Hyderabad, India

**Keywords:** dietary behavior, customised meal, responsible food consumption, multivariate logistic regression, educational institutes, SDGs

## Abstract

**Purpose:**

To determine the factors influencing the dietary habits of the varied groups among adults in India.

**Design/approach:**

Data on food habits such as choice of diet, preference toward meat, spicy food, sugar/calorie etc., were collected from the participants (from several higher education institutions) of different training programmes and events organised at ICAR-NAARM, Hyderabad and its students of Post Graduate Diploma in Agribusiness Management.

**Findings:**

Results of the study indicated that the food choice of the respondents is highly influenced by their region, age and gender. Most of the respondents preferred vegetarian food with increasing age. We also noticed that as age of the respondents increased, their preference toward simple & plain food (with less oil/spice) also increased. From the present investigation, it is recommended that the customized food menu should be prepared in every food serving institution based on the region, age and gender of the consumer.

**Novelty:**

Analysis of dietary patterns can be helpful for doctors, dieticians, food policy-making, restaurateurs, youth hostels, food organisations, mega kitchens etc. that would also contribute to responsible food consumption.

## 1. Introduction

Over the past few decades, people’s food preferences and dietary habits have shifted dramatically. People decide what to eat based on various criteria, scientifically defined as a “Diet.” The Analysis of dietary habits gives a more comprehensive impression of the food consumption habits within a population (1). Dietary pattern analysis, which summarises the entire diet, the foods, food categories, and nutrients contained, their combination and diversity, and the frequency and amount with which they are routinely ingested, has therefore been the focus of modern nutritional epidemiology investigations (2). For many individuals, the standard description of a food’s “taste” includes the chemical senses of style and olfaction (capacity of smelling) on which a person or group lives (3).

It is well known that people from different part of the country have different tastes for food. One of the explanations for various food preferences is that we have different experiences with food as we get older. Except for the formerly mentioned natural factors, most of our food preferences are learned through gests, and there are numerous ways of learning regarding food (4). It has been shown that with at first unlikeable food, intermittent exposure to new foods may increase feeling for that individual food (5). It is also systematically examined how different sugar statement forms influenced people’s perceptions of food categories (i.e., yoghurts, ice creams, cookies, and breakfast cereals) (6).

There is a growing interest in the analysis of dietary patterns of people for sustainable environmental development as the food habits of any individual is intrinsically connected to the human-food-environment chain. Good dietary habits may lead to a healthier life and longevity. Otherwise, it leads to unnecessary fat accumulation and severe health issues that negatively impact human health. A study on consent feeding revealed that people with high body fat percentages are highly vulnerable to impulsive dietary choices when compared with people with low body fat percentage people. This also increases the risk of obesity over time (7). Therefore, interventions in food taste regulations could help alter peoples’ evaluating of their food preferences, ultimately reducing the consumption of unhealthy foods (8). Nevertheless, following or adopting plant-based food with minimum consumption of meat products would also help contribute to environmental security through climate mitigation. Minimizing meat consumption and increasing healthy foods like vegetables and seafood helps reduce the Greenhouse Gases (GHGs) emission from the food production process besides meeting people’s health needs (9). The present work realises its significance because Indian food processing industries account for 32% of the country’s food market (10). Contrary to this belief, we must realise that 71% of the total Indian population self-identifies as non-vegetarian, though 39% eat non-veg occasionally, and 26% eat at least once a week (11). The United Nations Environment Programme (UNEP) (12) report brings out some fantastic facts: (i) The end consumer share of food waste share is about 61%, (ii) about 8–10% of GHGs emissions are associated with food that is not consumed.

**FIGURE 1:**
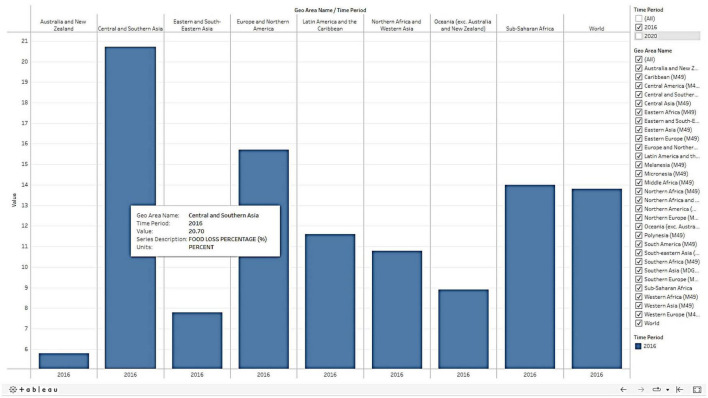
Food loss percentage in Central and South Asia (https://www.fao.org/sustainable-development-goals/indicators/1231/en/).

Higher education institutions and academies conduct various learning programs and trainings for which participants from different parts of the country are being invited. During the program, different types of food is being served to the participants based on the availability of ingredients in local market. Most of the times, many participants may not like food which is served during their stay at program venues. As a result, there will be huge wastage of food due to the dietary pattern of the participants as their region, age, gender etc. are highly influences the food consumption patterns. Day by day, food wastage reduction is becoming serious challenge for the administrators of the higher educational institutions. Changes in the food consumption pattern and planning the food menu based on the people’s preferences would significantly help in minimizing the food wastage. Nevertheless, food wastage is becoming a serious issue in the world. About 1.3 billion tonnes of food waste is produced yearly, approximately 1/3rd of global food production (13). As per the data of FAOSTAT, there is about 20.7% per capita food loss (Figure 1) in Central and South Asia. Therefore, United Nations’ sustainable development goal (SDG) 12.3 has set a target to halve the per capita global food waste and reduce food losses by 2030 (14). India is also holding many mega kitchens (Ex: Dharmasthala in Karnataka; Shirdi in Maharashtra; Golden Temple in Amritsar; Jagannath Temple in Puri; Kalinga Institute Kitchen Bhubaneswar) which serves the food for large number of people daily and also generates food wastage in large amount. Thus, it is the need of the hour to take appropriate actions toward reducing food wastage. Hence, changing our mindset on food preferences and reducing food waste plays a vital role in food sustainability.

We hypothesized that knowing the preference concerning age, gender, and regional diversity will help develop the most accepted food recipes, reduce food wastage, and improve the nutritional regime. Therefore, the present investigation aimed to determine the factors that impact the dietary habits of the varied groups among the Indian population.

## 2. Materials and methods

### 2.1. Dietary habits in India, vis-à-vis average around the world

**FIGURE 2:**
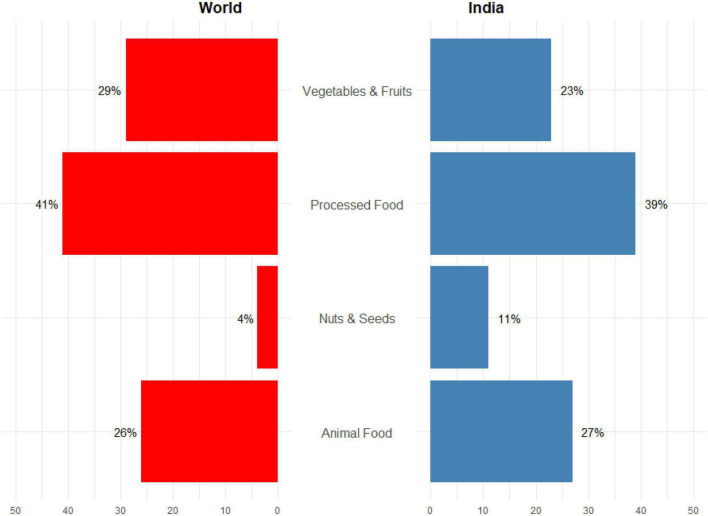
Components of food consumed by Indians and the world population (https://www.fao.org/3/ca9692en/online/ca9692en.html).

**FIGURE 3:**
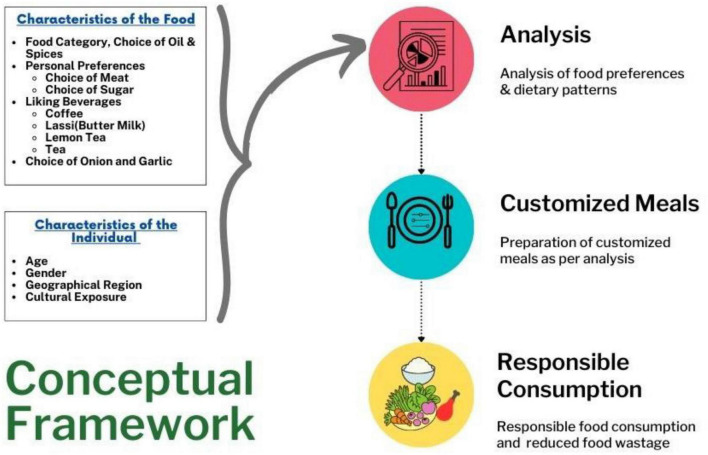
Conceptual framework of customised food planning.

There are some similarities and variations between the diets of Indians when compared with that of the world (Figure 2). One standout observation is that about a quarter of the Indian and world diet comprises animal produce. Similarly, almost 40% of the diets of the Indians and also the world altogether met from processed foods. Indians eat almost thrice (11%) of nuts and seeds, and the world population’s diet solely contains a stripped-down four-dimensional of nuts and seeds. Moreover, 23% of a typical Indian diet comprises vegetables and fruits, 6% less than the average global diet. Based on this assumption, we have conceptualized a framework of customized food planning (Figure 3) for responsible food consumption by analyzing the food preferences and dietary patterns.

### 2.2. Preparation of questionnaire and data collection

**FIGURE 4:**
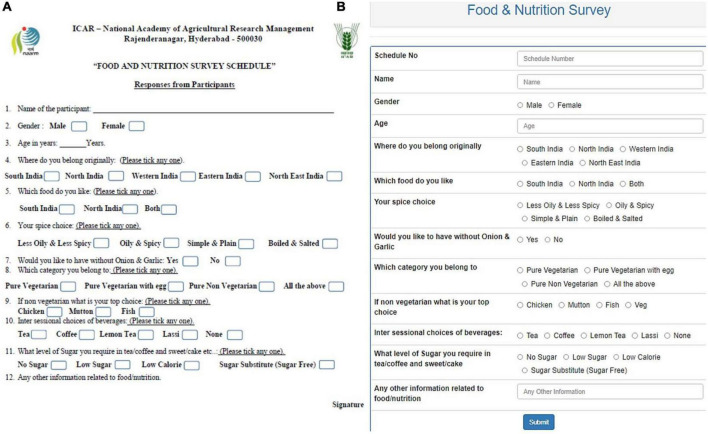
**(A)** Questionnaire on Food and Nutrition Survey Schedule. **(B)** Questionnaire portal.

**FIGURE 5:**
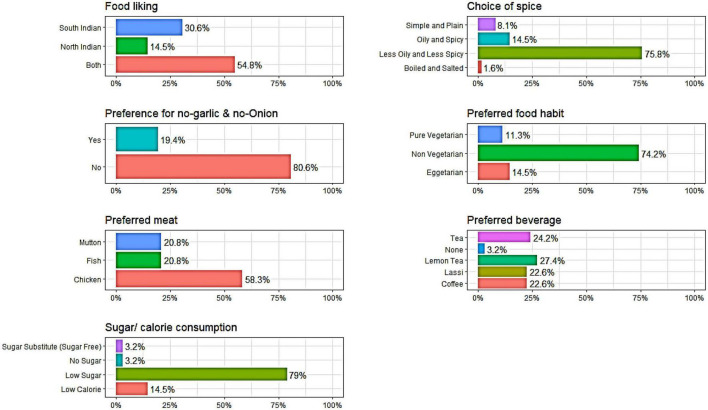
A food planning chart for the various programmes at ICAR-NAARM.

The data used in this study was collected through a structured questionnaire (Figure 4) from the Post Graduate Diploma in Agri Business Management (PGD-ABM) students and participants of different training programmes and events during 2018–2020 organised at ICAR-National Academy of Agricultural Research Management (NAARM), Hyderabad, India, which is an organisation under the Indian Council of Agricultural Research (ICAR), Ministry of Agriculture & Farmers Welfare, Govt. of India. The information related to the participants’/respondents’ food habits, such as their choice of diet, choice of meat, likeness toward spices, choice of beverages, garlic & ginger, sugar etc., was collected. In addition, respondents’ personal information, such as age, gender and their residing region, was also collected. All the variables under the study are categorical except age. The data was collected from 500 participants. The customised meals were served to the participants after administering the questionnaire and analysing the collected data. The food planning chart based on the food choice is depicted in Figure 5.

### 2.3. Statistical analysis

The influence of factors like age, gender and region of residence on food habits was studied using different appropriate statistical techniques. Since the variables are categorical, multinomial logistic regression was used to see the effect of these personal factors on dietary habits. Multinomial logistic regression is an extension of binary logistic regression, which helps predict when the dependent variable has three or more categories. The independent variables may be continuous or dichotomous (i.e., binary). Like binary logistic regression, multinomial logistic regression assesses the likelihood of categorical membership using maximum likelihood estimation (15). Out of 500 observations, 70% of the data was used for model building, and the remaining 30% was used for validating the developed model. Since there were seven predictor variables, each with *k* different number of classes, multinomial logistic regression equations were developed for each variable and *k-1* class. The personal variables such as gender, age and region of residence were considered predictor/independent variables, and the variables representing the dietary habits were considered response/dependent variables. The log odds values obtained from the multinomial logistic regression are presented in Table 2. A log odd values greater than zero indicates a positive association between the predictor variable and the outcome category, while a log odd less than zero indicates a negative association. The significance of the predictor variables is established by checking whether the log odds’ confidence interval includes zero. The accuracy percentage of the different multinomial logistic regression models built for training and validating data is presented in Table 3. The normalised chi-square test for association using Monte Carlo simulation of p-values was used to check for any association between response variables.

## 3. Results and discussion

### 3.1. Basic information about participants

Five hundred participants who responded to this study include students of Post Graduate Diploma in Agribusiness Management (PGD-ABM) of NAARM, probationary scientists and faculty from various higher education institutes in India. The respondents were aged between 19 and 59 years, with an average age of 30. About 60% of the respondents were males. The respondents from north India constituted the highest (about 57%).

### 3.2. Dietary behaviour and personal factors

Though the data was collected from 500 respondents, subjects with missing elements were ignored to avoid the problems of missing values. Accordingly, 14 records were expelled from the data, and the remaining 486 records were used for further statistical analysis. The different variables related to dietary behaviour and personal factors considered in the study are given in Table 1. The distribution of subjects based on the study variables is also given, along with whether they are used as a predictor or response variables in the multivariate logistic regression analysis. Regarding food likeliness, respondents liked both south and north Indian dishes. Most of the respondents preferred “less oily and less spicy” food, whereas the majority preferred dishes that included garlic and onion. Fifty per cent of the respondents identified themselves as preferring both vegetarian and non-vegetarian food. Among non-vegetarians, chicken was preferred by more respondents, followed by fish and mutton. Among the beverages, tea was preferred by most respondents, followed by lemon tea, coffee and lassi. Most respondents (66%) preferred low sugar in their food. The respondents in this study are well educated and have a good knowledge of food and nutrition that influence the dietary habit of an individual. Indeed, the level of education can influence food consumption patterns during adulthood (16).

**TABLE 1:** Study variables and distribution of subjects.

Variables	Classes	Frequency	Percentage	Variable type
Gender	Female	192	39.51	Predictor
	Male	294	60.49	
Age	Continuous variable	–	–	Predictor
Region	North India	276	56.79	Predictor
	South India	210	43.21	
Food liking	Both	282	58.02	Response
	North India	102	20.99	
	South India	102	20.99	
Choice of spice	Boiled and salted	13	2.67	Response
	Less oily & less spicy	317	65.23	
	Oily and spicy	64	13.17	
	Simple and plain	92	18.93	
Exclude onion and garlic.	No	407	83.74	Response
	Yes	79	16.26	
Food category	Non-vegetarian	102	20.99	Response
	Pure vegetarian	85	17.49	
	Eggetarian	54	11.11	
	All the above	245	50.41	
Choice of meat	Chicken	171	48.72	Response
	Fish	101	28.77	
	Mutton	79	22.51	
Choice of beverages	Coffee	96	19.75	Response
	Lassi	70	14.40	
	Lemon tea	134	27.57	
	None	15	3.09	
	Tea	171	35.19	
Sugar preference	Low calorie	108	22.22	Response
	Low sugar	320	65.84	
	No sugar	23	4.73	
	Sugar substitute (sugar-free)	35	7.20	

### 3.3. Food likeness

The multinomial logistic regression results presented in Table 2 show that the food likeness is influenced by age and the region of residence. As the age increases, it is observed that the preference changes from their regional dishes toward both. In fact, from an early age itself, taste and familiarity influence food preference. However, the preferences for tasty foods develop through experiences and are highly influenced by an individual’s attitude, beliefs and expectations (17). Regionality is also found to have a significant influence on food likeness. Log odds of likeness toward north Indian food (with reference class “both”) decreases by 2.54 if the respondent belongs to the southern region. Food intake habits and diet patterns will be significantly influenced by one’s culture and lifestyle (18, 19). One’s culture can strongly influence their dietary habit because of the peculiarity of different cultures’ food and cuisine (20). However, likeness toward south Indian food (with reference class “both”) increases by 3.13 log odds when the respondents belong to the southern region. This indicated that food choice is highly influenced by regionality. Gender was found not to influence food likeness. However, one of the studies conducted by Soam (21) on the organoleptic study of Hyderabadi Haleem (a type of food that made by cooking meat, wheat, barley or lentil in liquid for a long time) revealed that gender, regional and product familiarity influenced the acceptability of Haleem.

**TABLE 2:** Log odds values from the multinomial logistic regression.

Dependent variable	Reference class	Dependent class	Log odds (95% CI)			
			**Intercept**	**Gender** **(male)**	**Age**	**Region** **(South India)**
Food likeness	Both	North Indian	0.24 (–0.71, 1.19)	0.17 (–0.35, 0.68)	–0.03 (–0.06, –0.01)^#^	–2.54 (–3.48, –1.61)^#^
		South Indian	–1.61 (–2.94, –0.28)	–0.09 (–0.63, 0.45)	–0.06 (–0.09, –0.02)^#^	3.13 (2.27, 4)^#^
Choice of spice	Oily and spicy	Boiled & salted	0.32 (–3.97, 4.61)	0.08 (–1.5, 1.65)	–0.05 (–0.2, 0.1)	–0.8 (–2.31, 0.71)
		Less oily & Less spicy	1.15 (–0.53, 2.84)	–0.34 (–1.1, 0.42)	0.05 (–0.01, 0.1)	1.62 (1.07, 2.17)^#^
		Simple and plain	–0.87 (–2.71, 0.96)	–0.61 (–1.51, 0.28)	0.06 (0.02, 0.1)^#^	2.21 (–2.04, 2.38)^#^
Level of sugar	Low calorie	Low sugar	0.92 (–0.17, 2)	–0.23 (–0.79, 0.32)	0.01 (–0.02, 0.05)	–0.4 (–0.93, 0.12)
		No sugar	–5 (–7.07, –2.92)	–0.25 (–1.58, 1.09)	0.12 (0.06, 0.17)^#^	–0.85 (–2.1, 0.39)
		Sugar substitute (sugar-free)	–2.2 (–4.04, –0.37)	0.2 (–0.83, 1.23)	0.03 (–0.03, 0.08)	0.2 (–0.72, 1.13)
With or without onion/garlic	Yes	No	–2.29 (–3.36, –1.21)	–0.13 (–0.75, 0.49)	0.02 (–0.01, 0.05)	0.18 (–0.41, 0.77)
Category	All	Non-vegetarian	0.06 (–1.15, 1.28)	0.13 (–0.49, 0.75)	–0.03 (–0.07, 0.01)	–0.39 (–0.97, 0.19)
		Pure vegetarian	–0.95 (–2.02, 0.12)	–0.59 (–1.23, 0.04)	0.03 (–0.01, 0.06)	–1.16 (–1.82, –0.49)^#^
		Eggetarian	–1.7 (–3.11, –0.3)	–1.02 (–1.83, –0.2)^#^	0.03 (–0.01, 0.07)	–0.65 (–1.46, 0.15)
Choice of meat	Chicken	Fish	–1.27 (–2.43, –0.12)	–0.3 (–0.95, 0.34)	0.04 (0.01, 0.08)^#^	–0.45 (–1.05, 0.16)
		Mutton	–0.67 (–2.14, 0.8)	0.84 (0.05, 1.62)^#^	–0.02 (–0.07, 0.03)	–0.23 (–0.9, 0.44)
		Veg	–0.62 (–1.71, 0.47)	–0.63 (–1.24, –0.03)^#^	0.04 (0.01, 0.08)^#^	–1.16 (–1.76, –0.55)^#^
Choice of beverage	Coffee	Lassi	1.7 (–0.05, 3.46)	0.22 (–0.61, 1.05)	–0.06 (–0.12, 0)	–1.44 (–2.31, –0.57)^#^
		Lemon tea	0.39 (–0.83, 1.61)	0 (–0.67, 0.68)	0 (–0.03, 0.04)	–0.51 (–1.15, 0.13)
		None	–0.73 (–3.49, 2.03)	–0.32 (–1.6, 0.96)	–0.03 (–0.12, 0.06)	0.1 (–1.17, 1.36)
		Tea	0.9 (–0.26, 2.05)	0.15 (–0.49, 0.79)	0 (–0.04, 0.03)	–0.72 (–1.32, –0.11)^#^

^#^Indicates the significance of the log odds value since the confidence intervals do not contain zero.

### 3.4. Choice of spice

The choice of spice in food was found to be influenced by regionality. For example, if the respondents belonged to south India, the preference toward “simple & plain” food or “less oily and spicy” food (with reference class “oily & spicy”) increased by 2.21 and 1.62 log odds, respectively. Also, as the age of the respondents increased, their preference toward simple & plain food (with reference class “oily & spicy”) significantly increased by 0.06 log odds. In one of the studies, it is observed that youngsters are more likely to eat spicy food (22).

### 3.5. Level of sugar

The level of sugar was found to be influenced by the age of the respondents. As the age increased by one year, log odds of the preference toward no sugar (with reference class low calorie) increased by 0.12. Similar findings were reported by many other authors (23–25) and stated that high intake of free sugar is associated with poor diet, obesity and risk of non-communicable diseases such as diabetes and heart disease. Hence, it is advised that free sugar consumption should be reduced. Furthermore, people tend to avoid high-calorie foods which are high in sugar and salt as they age. For instance, one study showed that the elderly tend to restrict fast and nutrient-dense foods and adopt high-fibre and nutritious foods like fruits, vegetables and grains (26). Regionality was found to have no significant influence on the level of sugar.

### 3.6. With or without onion/garlic

Any of the determinant variables did not influence preference toward food with or without onion/garlic. This could be because many Indian traditions and Ayurveda suggest avoiding garlic and ginger as age increases. This is because it is believed that food, including garlic, meat, liquor and spices, brings out human behaviour’s lowest, crass qualities. In Indian culture, these items are considered Tamasika foods (27). However, recent research has demonstrated the benefits of garlic/onion and its extracts in various applications. This research suggested that the medicinal benefits of garlic and onions for several disorders may be returning. Garlic and onions include a variety of chemicals that are believed to lower the risk of cardiovascular disease, have anti-tumour and anti-microbial properties, and have benefits for high blood sugar levels (28). Therefore, eating garlic and onions should not be avoided.

### 3.7. Food category

Preference toward vegetarianism or non-vegetarianism was influenced by gender and regionality. If the respondent is male, the eggetarian decreased by 1.02 log odds (with reference class “all”). On the other hand, if the respondent belongs to the south Indian region, log the odds of preference toward pure vegetarian by 1.16 (with reference class “all”). The study conducted by Green et al. (29) showed that the intake of numerous food groups varied between studies in various parts of India. Dietary patterns from the east and south were also more likely to be characterised by meat or fish-eating than those from the north and west. They added that vegetarian diets are still typical in India and are associated with lower blood pressure. On the other hand, fruit, dairy products, and snacks were associated in a way that was favourably correlated with hypertension.

### 3.8. Choice of meat

The choice of meat was influenced by gender, age and regionality. For male respondents, log odds of the preference for mutton increased by 0.84, whereas the preference for vegetarian food decreased by 0.63 (with reference class “chicken”). As the age increased by one year, log odds of the preference toward fish and vegetarian food increased by 0.04 (with reference class “chicken”). This may be because as age increases, the efficiency of Gastro Intestinal System reduces to digest complex food like meat (30). Thus, aged respondents preferred vegetarian food and fish. Usually, red meat is rich in protein and filled with dense nutrients, and the gastrointestinal system plays a crucial role in digesting the same. Many studies reported that risk factors for chronic disease have shown that vegetarians have lower serum cholesterol concentrations, lower body mass indices, lower incidence of diabetes and possibly lower blood pressure than comparable non-vegetarians (31). Region-wise, log odds of the preference toward vegetarian food decreased by 1.16 if the respondent belonged to the South Indian region (with reference class “chicken”). Similar results were obtained by Green et al. (29), who, in their study, separated the models by region, year, age and sex in order to determine whether they showed a relationship with the specific food groups found in Indian dietary patterns. The consumption of many food groups varied across the studies from different regions. Sweets and snacks were more likely to characterise diets in the East and South, whereas fruit, vegetables, rice and pulses were more likely to characterise diets in the north and west. Dietary patterns from the East and South were also more likely to be defined by meat or fish consumption than those from the north and west.

### 3.9. Choice of beverages

The choice of beverages was found to be influenced by regionality. For respondents belonging to South India, coffee was most preferred. The log odds of preference toward Lassi and Tea decreased by 1.44 and 0.72 log odds (with reference to class “coffee”) for the respondents from south India. However, the model seems insufficient, indicating the influence of other factors not captured in the model (Table 3).

**TABLE 3:** Accuracy percentage for different multivariate logistic models.

Model for	Training data	Validation data
Food likeness	57.35	63.70
Choice of spice	64.12	66.44
Level of sugar	65.00	66.44
With or without garlic	82.94	85.62
Food category	50.88	46.58
Choice of meat	41.17	34.25
Choice of beverage	35.29	37.67

### 3.10. Association between response variables

The chi-square test was performed to check the association between different predictor variables in the study. The test’s null hypothesis is that there is no association between the two variables under consideration. The results of the chi-square test are given in Table 4. The results show a significant association at 1% level of confidence was found between food likeness and choice of spice; food likeness and category; food likeness and choice of beverage; choice of spice and choice of meat; and onion/garlic with category. At a 5% level of confidence, a significant association was observed between the choice of spice and onion/garlic; choice of spice and choice of beverages; onion/garlic and choice of meat; choice of meat and choice of beverage; choice of meat and level of sugar; and choice of beverage and level of sugar. All other variable combinations showed no significant association at 1 and 5% significance levels.

**TABLE 4:** Results of the normalised chi-square test for association.

	Choice of spice	With or without onion/garlic	Category (Veg/Non-veg/Veg + Egg/All)	Choice of meat	Choice of beverage	Level of sugar
Food likeness	29.009**	1.077	23.708**	34.818**	12.722	4.111
Choice of spice		8.676*	7.122	27.765**	22.211*	10.641
With or without onion/garlic			14.371**	9.307*	2.614	3.977
Category				NA	9.037	15.404
Choice of meat					22.811*	20.263*
Choice of beverage						22.908*

NA, not applicable.

*Significant at 5% level.

**Significant at 1% level.

### 3.11. Contribution to the reduction of greenhouse gas emissions and sustainable development goal

In this study, it is evident that as age increases, the consumption of meat-based foods decreases, and the preference toward vegetarian food has increased. Additionally, the respondents of higher education institutes were greatly concerned about healthy diets as their age increased. These kinds of healthy diet patterns among people significantly reduce food wastage and contribute to reducing greenhouse gas (GHGs) emissions. Therefore, food planning can be done considering the factors influencing food intake, which can help reduce food wastage. Reducing food wastage would directly reduce excess food production (that consumes more energy and releases more GHG to atmosphere), which minimises GHG emissions. In addition, minimizing meat consumption, adjusting the diet structure, and increasing the low carbon healthy foods like vegetables and seafood helps reducing the GHGs emission from the food production process besides meeting people’s health needs (9). They also revealed in their study that a plant-based diet could significantly reduce total GHG emissions by 41%. Usually, the meat production process releases large amounts of GHGs into the atmosphere. When the demand for meat production increases, the production process will gradually increase that results in emitting enormous concentrations of GHGs. A study conducted by Clune et al. (32) revealed that the production of beef per kilogram emits approximately 28.73 kg of carbon dioxide equivalent (CO_2_-eq), which is ten times more than that of rice production. This indicates that meat production releases more GHGs than that of plant production. Therefore, promoting low-carbon life by changing one’s diet structure would possibly reduce the GHG emissions that contribute to “SDG-13: *Climate Action*”. About 8–10% of GHGs are associated with food not being consumed, while target 12.3 of the Sustainable Development Goal (SDG) aims to reduce food wastage.

The EAT-LANCET commission paper published by Willett et al. (33) on healthy diets from sustainable food systems suggested the universally appropriate healthy diet, i.e., inclusion of legumes, nuts, unsaturated oils, whole grains, and fruits in the daily diet. Regarding non-vegetarian preferences, low to moderate seafood and poultry consumption was suggested. The study also addressed strictly lowering the consumption of red meat, processed meat and added sugar. Adopting this dietary plan in the individual’s lifestyle would help feed the global population of about 10 billion people a healthy diet within food production boundaries by 2050, besides achieving the SDG and Paris agreement.

### 3.12. Strengths and weakness of the study

The present study on the analysis of dietary trends is a novel approach to contribute immensely toward food menu planning in higher educational institutions. Indeed, the outcomes from the present investigation would be helpful for doctors, dieticians, food policy-makers, restaurateurs, mega kitchens, youth hostels, food organisations etc. For instance, the Kalinga Institute of Industrial Technology of Bhubaneshwar, the world’s largest tribal school, provides food to 25,000 students daily. It holds a mega kitchen, serves over 50,000 meals daily, and contributes to SDG #3 & #2 besides mitigating Global Hunger Index (GHI) through food planning. Thus, the present conceptualisation will help mega kitchens achieve the SDGs and GHI through proper food planning considering dietary preferences based on regionality, age and other choices.

However, to further improve the evaluation pattern of dietary-related studies, considering food wastage estimation, large-scale planning and analysis, increased sample size and inclusion of samples from multiple regions are crucial. In the present research, scientific food wastage estimations were not carried out because the customised meals were served after administering the questionnaire and analysing the collected data. As a result, there was no wastage observed.

## 4. Conclusion

In summary, our investigation revealed that the respondents of higher education institutes had a higher preference for a healthy diet with increasing age. Their preference for different food items was influenced by their regional differences, age, gender and other choices. We noticed that respondents from the south India region preferred simple and healthy food, while the north Indian respondents preferred both the south and north Indian dishes. The present study also reported that most respondents preferred vegetarian food with increasing age. In addition, most respondents preferred low-sugar food, indicating that knowledge of food and nutrition influence the dietary habit of an individual. The study also suggested proper food planning is required for higher educational institutions and mega kitchens where participants and stakeholders are from multiple regions. The present research findings can be helpful for menu planners at different institutes and programmes, including doctors, dieticians and researchers.

Conclusively, our research demonstrated that the food menu should be prepared based on the region, age, and gender of the consumer in any food-serving institution. So that zero-food wastage can be achieved through customised meal serving. In addition, appropriate food planning and management in higher educational institutions would immensely contribute to SDG and GHI, which helps reduce food wastage in India.

## Data availability statement

The raw data supporting the conclusions of this article will be made available by the authors, without undue reservation.

## Author contributions

SS performed the conceptualization, methodology, and supervision. SR and RB performed data collection. BY, RB, and RS performed the data curation. BY and RB performed software validation and formal analysis. SR, RB, SS, and BY performed writing—original draft. BY, RB, SR, TD, and BM carried out the literature survey and materials collection. SS, BY, TD, BM, SR, and RB carried out the review and editing. All authors read and approved the final manuscript and contributed to the study’s conception and design.
